# Influence of genetic variability at the surfactant proteins A and D in community-acquired pneumonia: a prospective, observational, genetic study

**DOI:** 10.1186/cc10030

**Published:** 2011-02-10

**Authors:** M Isabel García-Laorden, Felipe Rodríguez de Castro, Jordi Solé-Violán, Olga Rajas, José Blanquer, Luis Borderías, Javier Aspa, M Luisa Briones, Pedro Saavedra, J Alberto Marcos-Ramos, Nereida González-Quevedo, Ithaisa Sologuren, Estefanía Herrera-Ramos, José M Ferrer, Jordi Rello, Carlos Rodríguez-Gallego

**Affiliations:** 1 Department of Immunology, Hospital Universitario de Gran Canaria Dr. Negrín, Barranco de la Ballena s/n, Las Palmas de Gran Canaria, 35010, Spain; 2 Department of Respiratory Diseases, Hospital Universitario de Gran Canaria Dr. Negrín, Barranco de la Ballena s/n, Las Palmas de Gran Canaria, 35010, Spain; 3 Department of Medical and Surgical Sciences, School of Medicine, University of Las Palmas de Gran Canaria, Avenida Marítima del Sur s/n, Las Palmas de Gran Canaria, 35016, Spain; 4 Intensive Care Unit, Hospital Universitario de Gran Canaria Dr. Negrín, Barranco de la Ballena s/n, Las Palmas de Gran Canaria, 35010, Spain; 5 Department of Respiratory Diseases, Hospital Universitario de la Princesa, Diego de León 62, Madrid, 28005, Spain; 6 Intensive Care Unit, Hospital Clínico y Universitario de Valencia, Avenida Blasco Ibáñez 17, Valencia, 46010, Spain; 7 Department of Respiratory Diseases, Hospital San Jorge, Avenida Martínez de Velasco 36, Huesca, 22004, Spain; 8 Department of Respiratory Diseases, Hospital Clínico y Universitario de Valencia, Avenida Blasco Ibáñez 17, Valencia, 46010, Spain; 9 Department of Mathematics, University of Las Palmas de Gran Canaria, Campus Universitario de Tafira, Las Palmas de Gran Canaria, 35017, Spain; 10 Intensive Care Unit, Hospital Dr. José Molina Orosa, Carretera Arrecife-Tinajo km 1.300, Lanzarote, 35550, Spain; 11 Hospital Vall D'Hebron - Universitat Autonoma de Barcelona. CIBERES. Institut de Recerca Vall d'Hebron (VHIR), Passeig de la Vall d'Hebron 119-129, Barcelona, 08035, Spain

## Abstract

**Introduction:**

Genetic variability of the pulmonary surfactant proteins A and D may affect clearance of microorganisms and the extent of the inflammatory response. The genes of these collectins (*SFTPA1*, *SFTPA2 *and *SFTPD*) are located in a cluster at 10q21-24. The objective of this study was to evaluate the existence of linkage disequilibrium (LD) among these genes, and the association of variability at these genes with susceptibility and outcome of community-acquired pneumonia (CAP). We also studied the effect of genetic variability on SP-D serum levels.

**Methods:**

Seven non-synonymous polymorphisms of *SFTPA1*, *SFTPA2 *and *SFTPD *were analyzed. For susceptibility, 682 CAP patients and 769 controls were studied in a case-control study. Severity and outcome were evaluated in a prospective study. Haplotypes were inferred and LD was characterized. SP-D serum levels were measured in healthy controls.

**Results:**

The *SFTPD aa11-C *allele was significantly associated with lower SP-D serum levels, in a dose-dependent manner. We observed the existence of LD among the studied genes. Haplotypes *SFTPA1 6A*^*2 *^(*P *= 0.0009, odds ration (OR) = 0.78), *SFTPA2 1A*^*0 *^(*P *= 0.002, OR = 0.79), *SFTPA1-SFTPA2 6A*^*2*^*-1A*^*0 *^(*P *= 0.0005, OR = 0.77), and *SFTPD*-*SFTPA1-SFTPA2 C-6A*^*2*^*-1A*^*0 *^(*P *= 0.00001, OR = 0.62) were underrepresented in patients, whereas haplotypes *SFTPA2 1A*^*10 *^(*P = *0.00007, OR = 6.58) and *SFTPA1-SFTPA2 6A*^*3*^*-1A *(*P *= 0.0007, OR = 3.92) were overrepresented. Similar results were observed in CAP due to pneumococcus, though no significant differences were now observed after Bonferroni corrections. *1A*^*10 *^and *6A-1A *were associated with higher 28-day and 90-day mortality, and with multi-organ dysfunction syndrome (MODS) and acute respiratory distress syndrome (ARDS) respectively. *SFTPD aa11-C *allele was associated with development of MODS and ARDS.

**Conclusions:**

Our study indicates that missense single nucleotide polymorphisms and haplotypes of *SFTPA1, SFTPA2 *and *SFTPD *are associated with susceptibility to CAP, and that several haplotypes also influence severity and outcome of CAP.

## Introduction

Community-acquired pneumonia (CAP) is the most common infectious disease requiring hospitalization in developed countries. Several microorganisms may be causative agents of CAP, and *Streptococcus pneumoniae *is the most common cause [1]. Inherited genetic variants of components of the human immune system influence the susceptibility to and the severity of infectious diseases. In humans, primary immunodeficiencies (PID) affecting opsonization of bacteria and NF-κB-mediated activation have been shown to predispose to invasive infections by respiratory bacteria, particularly *S. pneumoniae *[2]. Conventional PID are mendelian disorders, but genetic variants at other genes involved in opsonophagocytosis, with a lower penetrance, may also influence susceptibility and severity of these infectious diseases with a complex pattern of inheritance [3].

In the lung, under normal conditions, microorganisms at first encounter components of the innate immune response, particularly alveolar macrophages, dendritic cells and the lung collectins, the surfactant protein (SP)-A1, -A2 and -D. SP-A1, -A2 and -D belong to the collectin subgroup of the C-type lectin superfamily, and contain both collagen-like and carbohydrate-binding recognition domains (CRDs) [4]. Upon binding to pathogen-associated molecular patterns (PAMPs), SP-A and SP-D enhance the opsonophagocytosis of common respiratory pathogens by macrophages [5,6]. Mice rendered SP-A or SP-D deficient exhibit increased susceptibility to several bacteria and viruses after intratracheal challenge [7-9]. SP-A1, -A2 and -D also play a pivotal role in the regulation of inflammatory responses [4,10,11] and clearance of apoptotic cells [4,12,13]. In mice, SP-A and SP-D have been shown to be non-redundant in the immune defense *in vivo *[9].

The human SP-A locus consists of two similar genes, *SFTPA1 *and *SFTPA2*, located on chromosome 10q21-24, within a cluster that includes the SP-D gene (*SFTPD*) [11]. The nucleotide sequences of human *SFTPA1 *and *SFTPA2 *differ little (96.0 to 99.6%) [14]. Single nucleotide polymorphisms (SNP) at the *SFTPA1 *codons 19, 50, 62, 133 and 219, and at the *SFTPA2 *codons 9, 91, 140 and 223 have been used to define the SP-A haplotypes, which are conventionally denoted as *6A*^*n *^for the *SFTPA1 *gene and *1A*^*n *^for the *SFTPA2 *gene (see Table E1 in Additional File 1) [15]. Variability at the *SFTPD *gene has been also reported. Particularly, the presence of the variant amino acid (aa)-11 (*M11T*) has been shown to lead to low SP-D levels [16].

In the present study, we assessed the potential association of missense polymorphisms of the *SFTPA1, SFTPA2 *and *SFTPD *genes as well as the resulting haplotypes, with the susceptibility to and the severity and outcome of CAP in adults. In addition, we evaluated the existence of linkage disequilibrium (LD) among these genes, and the effect of genetic variability on SP-D serum levels.

## Materials and methods

### Patients and controls

We studied 682 patients and 769 controls, all of them Caucasoid Spanish adult individuals from five hospitals in Spain. Foreigners and individuals with ancestors other than Spanish were previously excluded in the selection process. The diagnosis of CAP was assumed in the presence of acute onset of signs and symptoms suggesting lower respiratory tract infection and radiographic evidence of a new pulmonary infiltrate that had no other known cause. A detailed description of the exclusion criteria and clinical definitions are shown in Methods in Additional File 1[17-19]. The control group was composed of healthy unrelated blood donors from the same hospitals as patients.

For susceptibility, a case-control study was performed. Severity and outcome were evaluated in a prospective study of CAP patients. Demographic and clinical characteristics of CAP patients included in the study are shown in Table E2 in Additional File 1.

### Measurement of SP-D serum levels

In order to analyze the effect of the *SFTPD aa11 *on SP-D levels in our population, protein levels were measured in serum samples from individuals in the control group by means of a Surfactant Protein D ELISA kit (Antibodyshop^®^, Gentofte, Denmark).

### Genotyping

Four haplotypes of SP-A1 (*6A, 6A*^*2*^*, 6A*^*3 *^and *6A*^*4*^) and six of SP-A2 (*1A, 1A*^*0*^*, 1A*^*1*^*, 1A*^*2*^*, 1A*^*3 *^and *1A*^*5*^) are found frequently (>1%) in the general population [15]. On the basis of the differences in non-synonymous SNPs (*SFTPA1*-aa19, -aa50, -aa219, *SFTPA2*-aa9, -aa91, -aa223) the most frequent conventional haplotypes of these genes, except *1A *and *1A*^*5*^, can be unambiguously identified (see Table E1 in Additional File 1). However, this method does not allow for the differentiation of some of these haplotypes from those rare haplotypes (frequency equal or lower than 1%) identified with the SNPs indicated in Table E1 in Additional File 1. For comparative purposes, in our study each haplotype was denoted by the name of the most frequent haplotype for a given combination of non-synonymous SNPs. Genomic DNA was isolated from whole blood according to standard phenol-chloroform procedure or with the Magnapure DNA Isolation Kit (Roche Molecular Diagnostics, Pleasanton, CA, USA). Genotyping of polymorphisms in *SFTPA1 *(aa19, aa50, aa219), *SFTPA2 *(aa9, aa91, aa223) and *SFTPD *(aa11) genes was carried out using minor modifications of previously reported procedures [15,20]. The accuracy of genotyping was confirmed by direct sequencing in an ABI Prism 310 (Applied Biosystems, Foster City, CA, USA) sequencer.

Haplotypes for each individual were inferred using PHASE statistical software (version 2.1) [21]. The haplotype of *SFTPA1*, *SFTPA2 *or the haplotype encompassing *SFTPA1*, *SFTPA2 *and *SFTPD *was ambiguous or could not be assigned in 12 individuals, who were excluded from the study. The order used for the haplotypes nomenclature is *SFTPD-SFTPA1-SFTPA2*. Linkage disequilibrium (LD) was measured by means of Arlequin (version 3.11) [22] and Haploview [23] softwares in the control group. In addition, pairwise LD between haplotypes of *SFTPA1 *and *SFTPA2 *as well as with the *SFTPD *SNP was characterized using Arlequin 3.11. The existence of LD was considered if D' >0.4.

Informed consent was obtained from the patients or their relatives. The protocol was approved by the local ethics committee of the five hospitals. All steps were performed in complete accordance to the Helsinki declaration.

### Statistical analysis

Bivariate and multivariate statistical analyses were performed using SPSS (version 15.0) (SPSS, Inc, Chicago, Ill, USA) and R package [24]. A detailed description of the statistical methods is shown in Methods in Additional File 1.

## Results

### Susceptibility to CAP related to *SFTPA1*, *SFTPA2 *and *SFTPD *gene variants

Seven non-synonymous SNPs were genotyped across the region containing the *SFTPD*, *SFTPA1 *and *SFTPA2 *genes (Table 1). None of the SNPs showed a significant deviation from Hardy-Weinberg equilibrium in controls. Several major alleles were overrepresented in controls compared with patients, but only *SFTPA1 aa50-G, SFTPA2 aa9-A *and *aa91-G *remained significant after Bonferroni correction for multiple comparisons. A dominant effect of *SFTPA2 aa9-A*, and a recessive effect of *SFTPA1 aa50-G *and *aa219-C *as well as *SFTPA2 aa223-C *were associated with a lower risk of CAP (see Table 1).

**Table 1:** Comparison of SNPs from *SFTPD, SFTPA1 *and *SFTPA2 *between patients with CAP and controls

			Alleles comparison		**Genotypes comparison**^**†**^	
				
	Controls (N = 769)	CAP (N = 682)	P^*^	OR (95% CI)	P^*^	OR (95% CI)
*SFTPD *aa11 rs721917			*T vs C*		Dominant	
*T/T*	269 (35.0)	272 (39.9)			0.681	0.95 (0.73 to 1.1.23)
*T/C*	361 (46.9)	281 (41.2)	0.266	1.09 (0.94to 1.27)	Recessive	
*C/C*	139 (18.1)	129 (18.9)			0.054	1.23 (1.00 to 1.53)
*SFTPA1 *aa19 rs1059047			*T vs C*		Dominant	
*T/T*	680 (88.4)	582 (85.3)			0.193^‡^	0.22 (0.00 to 2.24)
*T/C*	88 (11.4)	96 (14.1)	0.056	0.75 (0.56 to 1.02)	Recessive	
*C/C*	1 (0.001)	4 (0.006)			0.081	0.76 (0.56 to 1.04)
*SFTPA1 *aa50 rs1136450			*G vs C*		Dominant	
*G/G*	320 (41.6)	232 (34.0)			0.060	0.77 (0.59 to 1.01)
*G/C*	330 (42.9)	319 (46.8)	0.002	0.79 (0.68 to 0.92)	Recessive	
*C/C*	119 (15.5)	131 (19.2)			0.003	0.72 (0.58 to 0.90)
*SFTPA1 *aa219 rs4253527			*C vs T*		Dominant	
*C/C*	620 (80.6)	508 (74.5)			0.710	1.24 (0.39 to 3.94)
*C/T*	142 (18.5)	169 (24.8)	0.012	0.75 (0.59 to 0.95)	Recessive	
*T/T*	7 (0.9)	5 (0.7)			0.005	0.70 (0.55 to 0.90)
*SFTPA2 *aa9 rs1059046			*A vs C*		Dominant	
*A/A*	323 (42.0)	245 (35.9)			0.010	0.68 (0.51 to 0.91)
*A/C*	349 (45.4)	318 (46.6)	0.003	0.79 (0.68 to 0.92)	Recessive	
*C/C*	97 (12.6)	119 (17.4)			0.018	0.77 (0.63 to 0.96)
*SFTPA2 *aa91 rs17886395			*G vs C*		Dominant	
*G/G*	623 (81.0)	501 (73.5)			0.110	0.58 (0.29 to 1.14)
*G/C*	133 (17.3)	158 (23.2)	0.0002	0.66 (0.52 to 0.82)	Recessive	
*C/C*	13 (1.7)	23 (3.4)			0.001	0.65 (0.51 to 0.83)
*SFTPA2 *aa223 rs1965708			*C vs A*		Dominant	
*C/C*	503 (65.4)	419 (61.4)			0.151	0.66 (0.38 to 1.17)
*C/A*	244 (31.7)	234 (34.3)	0.071	0.85 (0.70 to 1.02)	Recessive	
*A/A*	22 (2.9)	29 (4.3)			0.117	0.84 (0.68 to 1.04)

Frequency values are the number of individuals (%). SNPs: Single nucleotide polymorphisms; CAP: Community-acquired pneumonia.

* Uncorrected *P*-value for the bivariate comparison of alleles.

^† ^Uncorrected *P*-value for the bivariate comparison of genopytes. For the dominant allele effect, individuals homozygous for the more frequent allele or those heterozygous for both alleles were defined as 1, and individuals homozygous for the minor allele were defined as 0. For the recessive allele effect, individuals homozygous for the more frequent allele were defined as 1, with all others defined as 0.

^‡^*P*-value by Fischer exact test.

When haplotypes were inferred, seven different haplotypes were found for *SFTPA1 *and eight for *SFTPA2 *(see Table 2). All haplotypes except *6A*^*5*^*, 6A*^*15*^*, 1A*^*10 *^and *1A*^*13 *^had frequencies higher than 1% in our population. The most frequent haplotype for *SFTPA1 *and *SFTPA2 *were respectively *TGC *and *AGC*, which correspond mainly with the *6A*^*2 *^and *1A*^*0 *^haplotypes respectively. The frequencies of both haplotypes were significantly lower in patients compared to controls (*P *= 0.0009, OR = 0.78; 95% confidence interval (CI) 0.67 to 0.91, for *SFTPA1 6A*^*2*^. *P *= 0.002, OR = 0.79; 95% CI 0.68 to 0.92, for *SFTPA2 1A*^*0*^), even when Bonferroni correction was applied. Several haplotypes were overrepresented in patients compared with controls, but only *1A*^*10 *^(*P *= 0.00007, OR = 6.58; 95% CI 2.24 to 26.22) remained significant after Bonferroni correction. For the observed odd-ratios, the power of the tests with a significance level of 1% were 84.16%, 79.09% and 94.04% for the haplotypes *6A*^*2*^, *1A*^*0*^and *1A*^*10 *^respectively. In addition, dominant and recessive models showed a significant dominant effect on CAP susceptibility for haplotypes *6A*^*3*^, *1A*^*0*^, *1A*^*7 *^and *1A*^*10 *^and a recessive effect for haplotype *6A*^*2 *^(see Table 2).

**Table 2:** Comparison of haplotypes of *SFTPA1 *and *SFTPA2 *between patients with CAP and controls

Haplotype *	Controls N = 1,538	CAP N = 1,364	***P***^**†**^ OR (95% CI)	Haplotype effect	***P***^**‡**^ OR (95% CI)
*SFTPA1*					
6A (*CCC)*	75 (4.9)	90 (6.6)	0.047 1.38 (0.99-1.92)	Dominant	0.058 1.37 (0.99-1.91)
				Recessive	0.347^§ ^3.39 (0.27-178.36)
6A^2 ^*(TGC*)	934 (60.7)	745 (54.0)	0.0009 0.78 (0.67-0.91)	Dominant	0.172 0.83 (0.64-1.08)
				Recessive	0.0002 0.66 (0.53-0.82)
6A^3 ^(*TCC*)	362 (23.5)	343 (25.1)	n.s.	Dominant	0.004 1.37 ( (1.11-1.69)
				Recessive	0.146 1.35 (0.90-2.18)
6A^4 ^(*TCT*)	128 (8.3)	141 (10.3)	0.062 1.27 (0.98-1.65)	Dominant	0.068 1.28 (0.98-1.68)
				Recessive	0.726^§ ^1.66 (0.32-10.76)
6A^5 ^(*CCT*)	4 (0.3)	7 (0.5)	n.s.	Dominant	0.107 2.56 (0.78-8.34)
				Recessive	n.a.
6A^12 ^(*TGT*)	26 (1.7)	29 (2.1)	n.s.	Dominant	0.315 1.32 (0.77-2.28)
				Recessive	n.a.
6A^15 ^(*CGC*)	9 (0.6)	9 (0.7)	n.s.	Dominant	0.996 1.00 (0.39-2.61)
				Recessive	n.a.
*SFTPA2*					
*1A *(*CCC*)	134 (8.7)	147 (10.8)	n.s.	Dominant	0.050 1.31 (1.00-1.71)
				Recessive	0.80 1.13 (0.45-2.86)
*1A*^*0 *^(*AGC*)	911 (59.2)	729 (53.4)	0.002 0.79 (0.68-0.92)	Dominant	0.004 0.68 (0.52-0.88)
				Recessive	0.025 0.78 (0.62-0.97)
*1A*^*1 *^(*CGA*)	219 (14.2)	222 (16.3)	n.s.	Dominant	0.544 1.14 (0.91-1.44)
				Recessive	0.076 1.91 (0.925-3.93)
*1A*^*2 *^(*CGC*)	188 (12.2)	164 (12.0)	n.s.	Dominant	0.806 0.97 (0.76-1.24)
				Recessive	0.863 1.06 (0.53-2.12)
*1A*^*3 *^(*AGA*)	61 (4.0)	46 (3.4)	n.s.	Dominant	0.557 0.89 (0.59-1.33)
				Recessive	n.a.
*1A*^*7 *^(*ACC*)	21 (1.4)	32 (2.3)	0.049 1.74 (0.96-3.18)	Dominant	0.031 1.88 (1.05-3.36)
				Recessive	1.00^§ ^0.56 (0.01-10.84)
*1A*^*10 *^(*CCA*)	4 (0.3)	23 (1.7)	0.00007 6.58 (2.24-26.22)	Dominant	0.00006 6.68 (2.30-19.40)
				Recessive	n.a.
*1A*^*13 *^(*ACA*)	0	1 (0.1)	n.s.	Dominant	n.a.
				Recessive	n.a.

Frequency values are the number of chromosomes (%). CAP, Community-acquired pneumonia; n.s., non-significant; n.a., not assessable.

*Haplotypes for *SFTPA1 *and *SFTPA2*, resulting from the different combinations of the three SNPs (Single nucleotide polymorphisms) studied at each gene, are denoted using the conventional nomenclature [15].

^†^Uncorrected *P*-value for the bivariate comparison of haplotypes.

^‡^Uncorrected *P*-value for the bivariate comparison of genopytes. For the dominant haplotype effect, individuals homozygous or heterozygous for the allele of interest were defined as 1, and individuals without the haplotype were defined as 0. For the recessive haplotype effect, individuals homozygous for the haplotype of interest were defined as 1, with all others defined as 0.

^§^*P*-value by Fischer exact test.

### Linkage disequilibrium of *SFTPA1*, *SFTPA2 *and *SFTPD *genes

**Figure 1:**
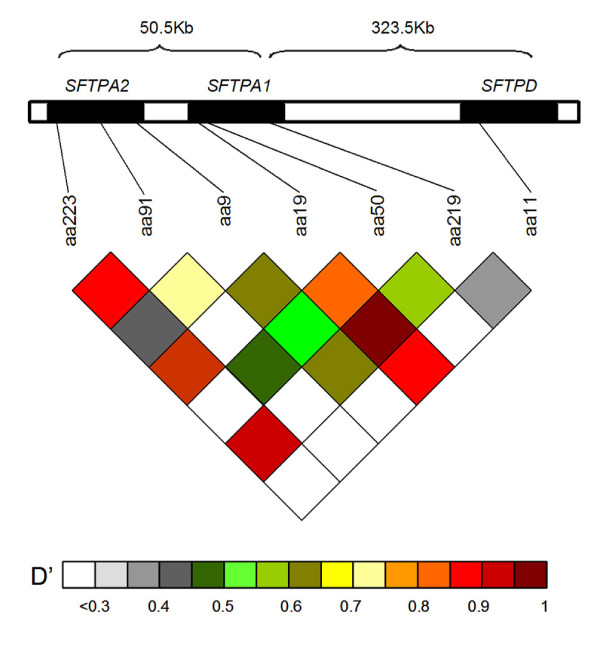
**Genomic organization, location of SNPs, and linkage disequilibrium (D') map for *SFTPD, SFTPA1 *and *SFTPA2 *genes**. SNPs: Single-nucleotide polymorphisms. All the D' values higher than 0.3 were statistically significant (*P *< 0.05). Linkage disequilibrium was measured in the control group.

Pairwise LD (D') measured by means of Arlequin confirmed the existence of LD among several SNPs at *SFTPA1 *and *SFTPA2*, whereas *SFTPD aa11 *was only observed in LD with *SFTPA1 aa19 *(see Figure 1). A similar pattern of LD was observed when D' was measured by means of the Haploview software (data not shown). *SFTPA1 *and *SFTPA2 *were previously found to be in LD [25,26]. The value of LD measured as r^2 ^was very low for every pair of SNPs (data not shown), and none of the studied SNPs could be used as haplotype-tagging SNP to infer the observed haplotypes.

When pairwise LD was measured among haplotypes instead among SNPs, *SFTPA1 *was found to be in LD with *SFTPD aa11*, but only a marginal LD was found between *SFTPA2 1A *and *SFTPD aa11 *(see Table E3 in Additional File 1).

### Susceptibility to CAP related to haplotypes encompassing *SFTPA1*, *SFTPA2 *and *SFTPD*

When haplotypes encompassing both *SFTPA *genes were studied, we observed 39 of the 64 expected haplotypes, and only 14 haplotypes had frequencies higher than 1% (data not shown). The most common *SFTPA1-SFTPA2 *haplotype, *6A*^*2*^*-1A*^*0*^, was underrepresented in patients (*P *= 0.0005, OR = 0.77; 95% CI 0.66 to 0.90), whereas *6A*^*3*^*-1A *was overrepresented (*P *= 0.0007, OR = 3.92; 95% CI 1.63 to 10.80) (see Table 3). Both differences remained significant after Bonferroni correction. For the observed odd-ratios, the powers of the tests with a significance level of 1% were 87.76% and 84.04% for the haplotypes *6A*^*2*^*-1A*^*0 *^and *6A*^*3*^*-1A *respectively. On the other hand, dominant and recessive logistic regression models showed a significant dominant effect on CAP susceptibility for haplotypes *6A*^*3*^*-1A *and *6A-1A*^*1 *^and a recessive effect for haplotype *6A*^*2*^*-1A*^*0 *^(see Table 3). We also intended to analyze whether phased variants encompassing the three genes were involved in susceptibility to CAP. Only 68 of the 128 expected haplotypes were observed, and 16 of them had a frequency over 1%. Chromosomes containing *C-6A*^*2*^*-1A*^*0 *^were decreased in patients when compared with controls (*P *= 0.00001, OR = 0.62; 95% CI 0.50 to 0.77), a difference that remained significant after Bonferroni correction. *C-6A*^*2*^*-1A*^*0 *^was also significantly associated with protection against CAP in a dominant model (see Table 3).

**Table 3:** Comparison of relevant haplotypes encompassing *SFTPD*, *SFTPA1 *and *SFTPA2 *between CAP patients and controls

Haplotype^*^	Controls	CAP	***P***^**†**^ OR (95% CI)	Haplotype effect	***P***^**‡**^ OR (95% CI)
*SFTPA1-SFTPA2*					
	N = 1538	N = 1,364			
*6A*^*2*^*-1A*^*0 *^(*TGCAGC*)	802 (52.1)	623 (45.7)	0.0005 0.77 (0.66-0.90)	Dominant	0.028 0.77 (0.61-0.97)
				Recessive	0.0005 0.65 (0.51-0.83)
*6A*^*3*^*-1A *(*TCCCCC*)	7 (0.5)	24 (1.8)	0.0007 3.92 (1.63-10.80)	Dominant	0.001 3.97 (1.70-9.27)
				Recessive	n.a.
*6A-1A*^*1 *^(*CCCCGA*)	2 (0.1)	9 (0.7)	0.020 5.10 (1.05-48.57)	Dominant	0.020 5.13 (1.10-23.82)
				Recessive	n.a.
*SFTPD-SFTPA1-SFTPA2*					
	N = 1,538	N = 1,364			
*C-6A*^*2*^*-1A*^*0 *^(*CTGCAGC*)	261 (17.0)	153 (11.2)	0.00001 0.62 (0.50-0.77)	Dominant	0.0001 0.63 (0.49-0.80)
				Recessive	0.003 0.38 (0.19-0.73)
*C-6A*^*3*^*-1A *(*CTCCCCC *)	3 (0.2)	14 (1.0)	0.003 5.31 (1.48-28.84)	Dominant	0.003 5.35 (1.53-18.70)
				Recessive	n.a.
*C-6A*^*4*^*-1A*^*2 *^(*CTCTTGC*)	15 (1.0)	31 (2.3)	0.005 2.36 (1.23-4.73)	Dominant	0.003 2.57 (1.35-4.87)
				Recessive	n.a.
*T-6A*^*3*^*-1A*^*1 *^(*TTCCCGA*)	54 (3.5)	74 (5.4)	0.012 1.58 (1.09-2.30)	Dominant	0.010 1.62 (1.12-2.34)
				Recessive	1.00 1.13^§ ^(0.01-88.64)
*T-6A*^*3*^*-1A*^*2 *^(*TTCCTGC*)	52 (3.4)	28 (2.1)	0.029 0.60 (0.36-0.97)	Dominant	0.019 0.57 (0.35-0.92)
				Recessive	n.a.

Frequency values are the number of chromosomes (%). CAP, Community-acquired pneumonia; n.a., not assessable.

*Haplotypes for *SFTPA1 *and *SFTPA2*, resulting from the different combinations of the three SNPs studied at each gene, are denoted using the conventional nomenclature [15].

^†^Uncorrected *P*-value for the bivariate comparison of haplotypes.

^‡^Uncorrected *P*-value for the bivariate comparison of genotypes. For the dominant haplotype effect, individuals homozygous or heterozygous for the haplotype of interest were defined as 1, and individuals without the haplotype were defined as 0. For the recessive haplotype effect, individuals homozygous for the haplotype of interest were defined as 1, with all others defined as 0.

^§^*P*-value by Fischer exact test.

A similar pattern of haplotype distribution was observed when individual as well as two- and three-gene based haplotypes were compared between pneumococcal CAP patients and healthy controls (see Table E4 in Additional File 1), though no significant differences were now observed after Bonferroni corrections.

### Outcome and severity of CAP patients related to genetic variants at *SFTPA1, SFTPA2 *and *SFTPD *genes

**Figure 2:**
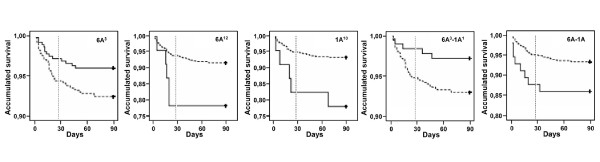
**Kaplan-Meier estimation of survival at 28 and 90 days in the 682 CAP patients**. CAP, community-acquired pneumonia. Solid curves represent the haplotypes under study, being dotted curves the rest of haplotypes. The vertical dotted line is depicted at 28 days. Significance levels for each comparison are shown in Table 4.

When fatal outcome was analyzed, patients who died within the first 28 days showed a higher frequency of haplotypes *6A*^*12*^*, 1A*^*10 *^and *6A-1A*, and a lower frequency of the major *SFTPA1aa19-T *and *aa219-C *alleles and of haplotypes *6A*^*3 *^and *6A*^*3*^*-1A*^*1 *^(see Table 4). Similar results were observed when 90-day mortality was analyzed (see Table 4). For the observed odd-ratios, the power of the tests with a significance level of 5% was 82.64% when the protective effect of *6A*^*3*^*-1A*^*1 *^on 28-day mortality was evaluated, and 81.45% and 80.79% concerning the effect of *6A*^*3 *^and *6A*^*3*^*-1A*^*1 *^on 90-day mortality respectively. Kaplan-Meier analysis (Figure 2) and log-rank test (Table 4) also showed significantly different survival for the above mentioned alleles and haplotypes. Cox Regression for 28-day survival, adjusted for age, gender, hospital of origin and co-morbidities, was significant for haplotypes *6A*^*12 *^and *6A-1A*, and it remained significant for haplotypes *6A*^*3 *^and *6A-1A *when 90-day survival analysis was performed (see Table 4). We also analyzed Cox Regression adjusted for hospital of origin, PSI and pathogen causative of the pneumonia, and we found similar results: for 28-day survival it remained significant for haplotype *6A-1A *(*P *= 0.029, OR = 2.45; 95% CI 1.10 to 5.46), although for *6A*^*12 *^haplotype it was not significant (*P *= 0.072); for 90-day survival it was significant for both *6A*^*3 *^(*P *= 0.038, OR = 0.52; 95% CI 0.28 to 0.96) and *6A-1A *(*P *= 0.045, OR = 2.12; 95% CI 1.02 to 4.44) haplotypes. No effect of the *SFTPD aa11 *SNP was observed. Due to the high number of observed haplotypes, and because of the limited sample size in the patient groups when they were stratified on the basis of severity and outcome, the haplotypes including *SFTPA1*, *A2 *and *D *were not studied.

**Table 4:** Outcome of CAP patients related to haplotypes of *SFTPA1 *and *SFTPA2*

	28 days					90 days				
		
	Mortality			Survival		Mortality			Survival	
Variant^*^	Yes	No	*P*^†^ OR (95% CI)	*P*^‡^ LR χ^2^	*P*^§^ HR (95% CI)	Yes	No	*P*^†^ OR (95% CI)	*P*^‡^ LR χ^2^	*P*^§^ HR (95% CI)
SNPs										
*SFTPA1 aa19-T *allele	58 (85.3)	1202 (92.7)	0.024 0.45 (0.22 to 1.03)	0.021 5.31	0.071 0.52 (0.25 to 1.06)	81 (88.0)	1179 (92.7)	0.105 0.58 (0.29 to 1.25)	0.091 2.85	0.256 0.68 (0.35 to 1.36)
*SFTPA1 aa219-C *allele	52 (76.5)	1133 (87.4)	0.009 0.47 (0.26 to 0.90)	0.009 6.75	0.085 0.57 (0.30 to 1.08)	72 (78.3)	1113 (87.5)	0.011 0.51 (0.30 to 0.92)	0.011 6.49	0.230 0.70 (0.39 to 1.25)
Haplotypes										
*SFTPA1*										
*6A*^*3*^	10 (14.7)	333 (25.7)	0.042 0.50 (0.22 to 1.00)	0.043 4.10	0.058 0.48 (0.23-1.02)	14 (15.2)	329 (25.9)	0.023 0.51 (0.27-0.93)	0.024 5.10	0.033 0.51 (0.28-0.95)
*6A*^*12*^	5 (7.4)	24 (1.9)	0.012^|| ^4.21 (1.21-11.74)	0.002 9.45	0.017 4.17 (1.29-13.46)	5 (5.4)	24 (1.9)	0.041^|| ^2.99 (0.87-8.25)	0.019 5.48	0.053 3.14 (0.98-10.03)
*SFTPA2*										
*1A*^*10*^	4 (5.9)	19 (1.5)	0.024^|| ^4.20 (1.01-13.13)	0.005 7.92	0.401 1.85 (0.44-7.79)	5 (5.4)	18 (1.4)	0.016^|| ^4.00 (1.13-11.52)	0.003 8.93	0.275 1.92 (0.59-6.23)
*SFTPA1-SFTPA2*										
*6A*^*3*^*-1A*^*1*^	3 (4.4)	163 (12.6)	0.045 0.32 (0.06-1.00)	0.047 3.94	0.063 0.26 (0.06-1.08)	5 (5.4)	161 (12.7)	0.041 0.40 (0.12-0.98)	0.043 4.40	0.055 0.373 (0.14-1.02)
*6A-1A*	7 (10.3)	51 (3.9)	0.022^|| ^2.80 (1.03-6.55)	0.008 6.93	0.024 2.66 (1.14-6.30)	8 (8.7)	50 (3.9)	0.053^|| ^2.33 (0.92-5.16)	0.021 5.31	0.045 2.23 (1.02-4.89)

Frequency values are the number of chromosomes (%). Only relevant haplotypes are shown. SNPs: Single nucleotide polymorphisms; CAP: Community-acquired pneumonia.

*Haplotypes for *SFTPA1 *and *SFTPA2*, resulting from the different combinations of the three SNPs studied at each gene, are denoted using the conventional nomenclature [15].

^†^*P *value for the bivariate comparison.

^‡^*P *value for log-rank (LR) χ^2 ^test for survival rates related to haplotypes.

^§^*P *value for Cox proportional hazard ratio for multivariate analysis, including the variables age, gender, hospital of origin and co-morbidities.

^||^*P *value by Fischer exact test.

The relevance of these genetic variants in the severity of CAP was also evaluated by analyzing predisposition to acute respiratory distress syndrome (ARDS) and to multi-organ dysfunction syndrome (MODS) (see Tables 5 and 6). The *SFTPD aa11-C *allele was significantly overrepresented in patients with MODS or ARDS. Haplotypes *6A *and *6A-1A*, were also associated with the development of ARDS, and *SFTPA2 1A *and *1A*^*10 *^were associated with the development of MODS. For the observed odd-ratios, the power of the association of *1A *with predisposition to MODS was 89.29%. However, the number of individuals included in the analysis of outcome was relatively small and the power of the tests with a significance level of 1% was lower than 80%. These associations remained significant in multivariate analysis adjusted for age, gender, hospital of origin and co-morbidities, as well as for hospital of origin, PSI and causative microorganism (see Tables 5 and 6). By contrast, *6A*^*3*^*-1A*^*1 *^was associated with protection against MODS, although this difference was not significant in the multivariate analysis.

**Table 5:** Predisposition to MODS related to *SFTPD *alleles and to *SFTPD, SFTPA1 *and *SFTPA2 *haplotypes in patients with CAP

Allele or haplotype*	MODS	No MODS	***P***^**†**^ OR (95% CI)	***P***^**‡**^ OR (95% CI)	***P***^**§**^ OR (95% CI)
*SFTPD*	N = 178	N = 1,186			
*C*	85 (47.8)	454 (38.4)	0.016 1.47 (1.06-2.05)	0.002 1.68 (1.20-2.35)	0.043 1.46 (1.01-2.10)
*SFTPA1*	N = 178	N = 1,186			
*6A*	14 (7.9)	76 (6.4)	0.465 1.25 (0.64-2.29)	-	-
*SFTPA2*	N = 178	N = 1,186			
*1A*	32 (18.0)	115 (9.7)	0.0009 2.04 (1.28-3.17)	0.0004 2.29 (1.45-3.62)	0.002 2.21 (1.34-3.65)
*1A*^*10*^	8 (4.5)	15 (1.3)	0.006^||^ 3.67 (1.33-9.38)	0.033 2.70 (1.08-6.76)	0.033 2.98 (1.09-8.10)
*SFTPA1-SFTPA2*	N = 178	N = 1,186			
*6A-1A*	12 (6.7)	46 (3.9)	0.078 1.79 (0.85-3.52)	-	-
*6A*^*3*^*-1A*^*1*^	13 (7.3)	153 (12.9)	0.033 0.53 (0.27-0.97)	0.115 0.62 (0.34-1.13)	0.097 0.58 (0.31-1.10)

For allelic and haplotypic frequencies values are the number of chromosomes (%). Only relevant haplotypes are shown. CAP: Community Acquired Pneumonia; MODS: Multi-organ Dysfunction Syndrome.

*Haplotypes for *SFTPA1 *and *SFTPA2*, resulting from the different combinations of the three SNPs (Single nucleotide polymorphisms) studied at each gene, are denoted using the conventional nomenclature [15].

^†^*P*-value for the bivariate comparison.

^‡^*P*-value for multivariate analysis, including the variables age, gender, hospital of origin and co-morbidities. For those bivariate comparisons that resulted in non-significant differences, multivariate analysis were not calculated.

^§^*P*-value for multivariate analysis, including the variables hospital of origin, PSI (Pneumonia Severity Index) and pathogen.

^||^*P*-value by Fischer exact test.

**Table 6:** Predisposition to ARDS related to *SFTPD *alleles and to *SFTPD, SFTPA1 *and *SFTPA2 *haplotypes in patients with CAP

Allele or haplotype *	ARDS	No ARDS	***P***^**†**^ OR (95% CI)	***P***^**‡**^ OR (95% CI)	***P***^**§**^ OR (95% CI)
*SFTPD*	N = 52	N = 1,312			
*C*	29 (55.8)	510 (38.9)	0.015 1.98 (1.09-3.63)	0.032 1.92 (1.06-3.48)	0.050 1.79 (1.00-3.20)
*SFTPA1*	N = 52	N = 1,312			
*6A*	8 (15.4)	82 (6.3)	0.018^||^ 2.73 (1.07-6.11)	0.004 3.89 (1.56-9.72)	0.022 2.64 (1.15-6.08)
*SFTPA2*	N = 52	N = 1,312			
*1A*	7 (13.5)	140 (10.7)	0.524 1.30 (0.49-2.98)	-	-
*1A*^*10*^	1 (1.9)	22 (1.7)	0.594^||^ 1.15 (0.03-7.40)	-	-
*SFTPA1-SFTPA2*	N = 52	N = 1,312			
*6A-1A*	7 (13.5)	51 (3.9)	0.005^§^ 3.85 (1.39-9.15)	0.0006 5.83(2.12-16.04)	0.012 3.16 (1.28-7.80)
*6A*^*3*^*-1A*^*1*^	5 (9.6)	161 (12.3)	0.566 0.76 (0.23-1.94)	-	-

For allelic and haplotypic frequencies values are the number of chromosomes (%). Only relevant haplotypes are shown. CAP: Community Acquired Pneumonia; ARDS: Acute Respiratory Distress Syndrome.

*Haplotypes for *SFTPA1 *and *SFTPA2*, resulting from the different combinations of the three SNPs (Single nucleotide polymorphisms) studied at each gene, are denoted using the conventional nomenclature [15].

^†^*P *value for the bivariate comparison.

^‡^*P *value for multivariate analysis, including the variables age, gender, hospital of origin and co-morbidities. For those bivariate comparisons that resulted in non-significant differences, multivariate analysis were not calculated.

^§^P value for multivariate analysis, including the variables hospital of origin, PSI (Pneumonia Severity Index) and pathogen.

^||^*P*^-^value by Fischer exact test.

### Association of genetic variants at *SFTPD *with serum levels of SP-D

**Figure 3:**
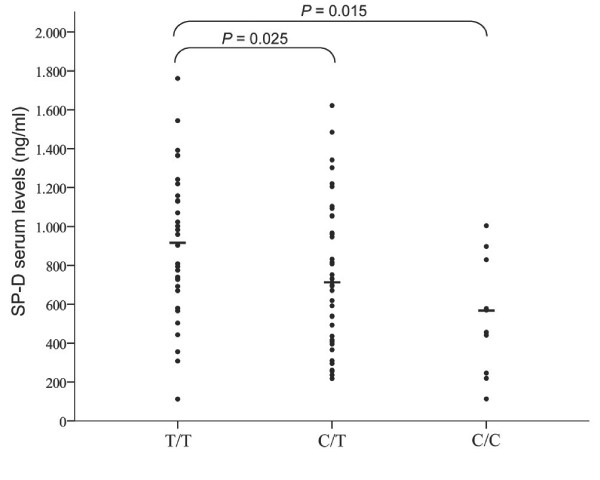
**SP-D serum levels (ng/ml) regarding to *SFTPD *genotypes in healthy controls**. The comparison of the three groups showed a significant difference (ANOVA *P *= 0.017). Horizontal lines denote mean value for each genotype.

In order to study whether variants at the pulmonary collectins were associated with differences of serum levels of SP-D, this protein was measured in serum from healthy controls with known genotypes. The *SFTPD aa11-C *SNP associated with lower SP-D serum levels (905.10 ± 68.38 ng/ml for *T/T *genotype, 711.04 ± 52.02 ng/ml for *T/C*, and 577.91 ± 96.14 ng/ml for *C/C*; ANOVA *P *= 0.017) (see Figure 3).

## Discussion

This study is unique in reporting a genetic association between non-synonymous SNPs at *SFTPD*, *SFTPA1 *and *SFTPA2*, as well as of haplotypes encompassing these genes, with the susceptibility, severity and outcome of CAP.

The major alleles of *SFTPA1 aa50-G, aa219-C as well as SFTPA2 aa9-A *and *aa91-G *or genotypes carrying these alleles were associated with protection against CAP. The frequencies of the different SNPs and haplotypes of *SFTPA1, SFTPA2 *and *SFTPD *observed in our study were similar to those previously reported in European populations [25]. *SFTPA1 *and *SFTPA2 *were reported to be in strong LD [26,27], and several haplotypes of these loci tend to segregate together, being *6A*^*2*^*-1A*^*0 *^the major haplotype [27]. A protective role against CAP was associated with *6A*^*2*^, *1A*^*0 *^and *6A*^*2*^*-1A*^*0 *^in our survey but only the rare *1A*^*10 *^and *6A*^*3*^*-1A *haplotypes were significantly associated with susceptibility to CAP. Similar results were observed in susceptibility to pneumococcal CAP. Several SNPs and haplotypes were also associated with a higher severity and poor outcome; MODS, ARDS, and mortality were selected because they represent the more severe clinical phenotypes. Particularly, *1A*^*10 *^and *6A-1A *were overrepresented among patients who died at 28 or 90 days, and they also predisposed to MODS and ARDS respectively. Likewise, *6A *was associated with ARDS, and *1A *was associated with MODS. By contrast, *6A*^*3 *^and *6A*^*3*^*-1A*^*1 *^were underrepresented in patients who died. The *SFTPD aa11-C *allele was associated with the development of MODS and ARDS, but no significant effects on mortality were observed. In spite that the power of the test for some associations with outcome and severity were higher than 80% for the observed OR with a significance level of 5%, the number of individuals included in the analysis of outcome was relatively small. Consequently, associations with outcome should be interpreted with caution.

Only a few studies have addressed the role of the genetic variability at *SFTPA1*, and *SFTPA2 *in infectious diseases [28-31]. In bacterial infections, homozygosity for the *1A*^*1 *^haplotype was reported to be associated with meningococcal disease [30]. Noteworthy, *6A*^*2*^*-1A*^*0 *^was protective against acute otitis media (AOM) in children [32]. Haplotypes *6A*^*2 *^and *1A*^*0 *^may also be involved in protection against respiratory syncytial virus (RSV) disease [29,33]. Considering the high difference in the frequencies with the corresponding alternative alleles and haplotypes, it is tempting to speculate that *6A*^*2*^, *1A*^*0 *^and *6A*^*2*^*-1A*^*0 *^could have been maintained at high frequencies partly by their protective effect against respiratory infections. The *6A *and *6A-1A *haplotypes were found to be associated with an increased risk of wheeze and persistent cough, presumably triggered by respiratory infections or environmental contaminants, among infants at risk for asthma [27]. Regarding SP-D, the *SFTPD aa11-T *allele was associated with severe RSV bronchiolitis [34], whereas the *SFTPD aa11-C *variant was associated with tuberculosis [30].

In sharp contrast to the potentially proinflammatory effects after PAMP recognition by collectins, mice deficient in SP-A or SP-D develop enhanced inflammatory pulmonary responses [35-37]. SP-A and SP-D play a dual role in the inflammatory response. They interact with pathogens via their CRD, and are recognized by calreticulin/CD91 on phagocytes through the N-terminal collagen domain, promoting phagocytosis and proinflammatory responses [10,13]. By contrast, binding of the CRD to signal inhibitory regulatory protein α (SIRPα) on alveolar macrophages suppresses NF-κB activation and inflammation, allowing the lung to remain in a quiescent state during periods of health [10]. A similar dual effect is observed in the promotion or inhibition of apoptosis [12]. SP-A and SP-D can also inhibit inflammation by blocking, through the CRD, Toll-like receptors 2 and 4 [38,39]. In agreement with previous results [16], we have observed that the *SFTPD aa11-C *allele associates with significantly lower SP-D serum levels than the *aa11-T *allele, and this effect was dose-dependent. The *aa11-C/T *SNP, located in the N-terminal domain, influences oligomerization of SP-D and explains a significant part of the heritability of serum SP-D levels [16,40]. Serum from *aa11-C *homozygotes lack the highest molecular weight (m.w.) forms of the protein, which binds preferentially to complex microorganisms whereas the low m.w. SP-D preferentially binds LPS [16].

As a consequence of intracellular oligomerization, monomeric SP-A subunits fold into trimers, and supratrimeric assembly leads to high-order oligomers [41,42]. The degree of supratrimeric oligomerization is important for the host defense function [14,41,43-45]. SP-A1 and SP-A2 differ in only four amino acids (residues 66, 73, 81 and 85) located in the collagen domain [46]. In most functions examined, recombinant human (rh) SP-A2 shows higher biological activity than SP-A1 [14,41,47-50].

The significance and the nature of functional differences between variants at SP-A1 and SP-A2 are poorly understood [14,49,50]. Variants *aa50 *(SP-A1) and *aa91 *(SP-A2) are located in the collagen region. These changes may affect the oligomerization pattern and binding to receptors such as calreticulin/CD91 or the functional activity of the protein. Likewise, the variants *aa219 *(SP-A1) and *aa223 *(SP-A2) are located in the CRD, and might directly influence the binding properties to microorganisms or to surface receptors such as SIRPα or TLR4. Residue 9, and frequently residue 19, is located in the signal peptide, and it is not know whether these variants may affect the function of the protein [14,44]. Alternatively all the missense variants could be in LD with SNPs in regulatory regions that might affect translation and RNA stability [51,52].

Native SP-A is thought to consist of hetero-oligomers of SP-A1 and SP-A2, and properties of co-expressed SP-A1/SP-A2 are between those of SP-A1 and SP-A2 [41,46]. However, the extent of oligomerization of SP-A, as well as the SP-A1/SP-A2 ratio, may be altered in various diseases and can vary among individuals [53,54]. The combination of both gene products may be important for reaching a fully native conformation [41]. In fact, it was recently shown that both SP-A1 and SP-A2 are necessary for the formation of pulmonar tubular myelin [55]. Therefore, the effect of a given haplotype may be largely influenced by haplotypes at the other gene. Our results suggest that the *6A*^*2 *^*to1A*^*0 *^haplotype is more protective against CAP than both *6A*^*2 *^and *1A*^*0*^.

It was previously reported that the *SFTPD aa11 *SNP is in LD with *SFTPA1 *and *SFTPA2 *[25]. A protective effect of the *6A*^*2 *^*to 1A*^*0 *^haplotype was even higher when this haplotype co-segregates with the *SFTPD aa11-C *allele. Likewise, one haplotype containing *6A*^*2*^*-1A*^*0 *^and the *G *allele of the *SFTPD aa160 *SNP could be protective against severe RSV disease [29]. Haplotypes at *SFTPA1 *are in LD with *SFTPD aa11 *in our population, but only a marginal LD between *SFTPA2 *and *SFTPD aa11 *was observed. In addition, no LD between *6A*^*2 *^*to A*^*0 *^and *SFTPD aa11 *was found in controls (D' = 0.09) or CAP patients (D' = 0.024) in our study. These findings suggest that the protective effect of the co-segregation of *SFTPD aa11-C *with *6A*^*2 *^*to 1A*^*0 *^on CAP susceptibility may rather reflect genetic interactions. Alternatively, the *SFTPD aa11 *SNP may be a marker of other SNPs in LD with *SFTPA1 *and *SFTPA2*. The gene of another collecting, the mannose-binding lectin (MBL), is located at 10q11.2-q21. We have previously observed that MBL deficiency predisposes to higher severity and poor outcome in CAP [56], and LD of the SP genes with *MBL2 *cannot be ruled out.

Despite modern antibiotics, CAP remains a common cause of death, and the search for new therapeutic approaches has been redirected into non-antibiotic therapies [57]. SP-A levels are reduced in several pulmonary diseases [58-60]. SP-D may also be reduced in some patients with ARDS [59]. In *Sftpa*^*-/- *^and *Sftpd*^*-/- *^mice, intratracheally administered SP-A or SP-D can restore microbial clearance and inflammation [8,35]. Exogenous surfactant preparation containing the hydrophobic SP-B and -C are nowadays widely used for replacement therapies in infantile RDS. In addition, intratracheal instillation of recombinant SP-C reduced mortality in patients with severe ARDS due to pneumonia or aspiration [61]. Some of the genetic variants analyzed in our survey, such as *1A*^*10*^, although rare, may have a high impact on susceptibility, severity and outcome of CAP. Validation of our results in other populations, and a better knowledge of the functional and clinical significance of the genetic variability at *SFTPA1*, *SFTPA2 *and *SFTPD *could be relevant for future investigations in the use of these collectins in the treatment of respiratory infectious diseases.

## Conclusions

The surfactant proteins A1, A2 and D are key components of innate immune response and the anti-inflammatory status in the lung. Genetic variability at the genes of these collectins influences susceptibility and outcome of community-acquired pneumonia. These results could be relevant for future investigations in the use of these collectins in the treatment of respiratory infectious diseases.

## Key messages

• The *SFTPA1 *and *SFTPA2 *haplotypes *6A*^*2*^, *1A*^*0 *^and *6A*^*2 *^*to 1A*^*0*^, and the *SFTPD-SFTPA1-SFTPA2 *haplotype *C-6A*^*2 *^*to 1A*^*0 *^are associated with a protective role against the development of Community-acquired pneumonia (CAP).

• *1A*^*10 *^and *6A*^*3 *^*to 1A *haplotypes are associated with increased susceptibility to CAP.

• Haplotypes *6A *and *6A to 1A *are associated with development of ARDS, while *1A *and *1A*^*10 *^are associated with MODS in patients with CAP.

• The variant *SFTPD aa11-C *leads to decreased SP-D serum levels, and predisposes to development of MODS and ARDS in patients with CAP.

• Haplotypes *6A*^*12*^, *1A*^*10 *^and *6A to 1A *are overrepresented among patients who died at 28 or 90 days. By contrast, *6A*^*3 *^and *6A*^*3 *^*to 1A*^*1 *^are protective against 28-day and 90-day mortality.

## Abbreviations

AOM: acute otitis media; ARDS: acute respiratory distress syndrome; CAP: community-acquired pneumonia; CRD: carbohydrate-binding recognition domain; LD: linkage disequilibrium; MBL: mannose-binding lectin; MODS: multi-organ dysfunction syndrome; PAMP: pathogen-associated molecular pattern; PID: primary immunodeficiency; RSV: respiratory syncitial virus; SIRP: signal inhibitory regulatory protein; SNP: single nucleotide polymorphism; SP: surfactant protein; TLR: toll-like receptor.

## Competing interests

The authors declare that they have no competing interests.

## Authors' contributions

MIGL did the genotyping and protein measurements, analyzed and interpreted the data, and contributed to the writing of the manuscript. FRC and JSV were responsible for the clinical evaluations of patients, samples and data collection, collaborated in designing the study, as well as contributed to the interpretation of data and the writing of the manuscript. OR, JB, LB, JA, MLB, JAMR, JMF and JR were also responsible for clinical evaluation of patients, samples and data collection. PS participated in the statistical analysis. NGQ, IS and EHR did genotyping. CRG conceived the study, analyzed and interpreted data, and wrote the manuscript.

## Supplementary Material

Additional file 1 **Further description of methods, definitions and statistical analysis, and Tables E1-E4**. The file contains additional information on exclusion criteria and definitions of PSI, ARDS and MODS. The statistical tests used are described. The additional file also includes four tables. Table E1 defines the resulting haplotypes from SNPs combination in *SFTPA1 *and *SFTPA2 *genes. Table E2 presents demographic and clinical characteristics of CAP patients. Table E3 shows the pairwise linkage disequilibrium measure for surfactant proteins A1, A2 and D alleles. Table E4 compares haplotypes of *SFTPA1, SFTPA2 *and *SFTPD *between patients with pneumococcal CAP and controls. Click here for file
