# Supplementary material for: Influence of genetic variability at the surfactant proteins A and D in community-acquired pneumonia: a prospective, observational, genetic study
Source: Crit Care. 2011 Feb 10;15(1):R57. doi: 10.1186/cc10030 (PMC3221990; doi:10.1186/cc10030)
Supplement: Additional file 1 — Further description of methods, definitions and statistical analysis, and Tables E1-E4. The file contains additional information on exclusion criteria and definitions of PSI, ARDS and MODS. The statistical tests used are described. The additional file also includes four tables. Table E1 defines the resulting haplotypes from SNPs combination in SFTPA1 and SFTPA2 genes. Table E2 presents demographic and clinical characteristics of CAP patients. Table E3 shows the pairwise linkage disequilibrium measure for surfactant proteins A1, A2 and D alleles. Table E4 compares haplotypes of SFTPA1, SFTPA2 and SFTPD between patients with pneumococcal CAP and controls. [file cc10030-S1.DOC]

**Supplementary material**

**Influence of genetic variability at the surfactant proteins A and D in community-acquired pneumonia: a prospective, observational, genetic study.**

M. Isabel García-Laorden, Felipe Rodríguez de Castro, MD, Jordi Solé-Violán, Olga Rajas, José Blanquer, Luis Borderías, Javier Aspa, M. Luisa Briones, Pedro Saavedra, J. Alberto Marcos-Ramos, Nereida González-Quevedo, Ithaisa Sologuren, Estefanía Herrera-Ramos, José M. Ferrer, Jordi Rello, Carlos Rodríguez-Gallego.

**METHODS**

**Patients and controls.**

Patients with defined severe immunosuppression or HIV positivity, as well as patients with tuberculosis, obstructive pneumonia caused by neoplasia or pneumonia as terminal event of chronic and progressive disease, were excluded from the study.

The pneumonia severity index (PSI) was measured using the Fine scale [17]. Acute respiratory distress syndrome (ARDS) was defined using the American European Consensus Conference Definition [18]. Multi-organ dysfunction syndrome (MODS) was defined using the American College of Chest Physicians/Society of Critical Care Medicine criteria [19]. A diagnosis of pneumococcal pneumonia was considered with one of the following criteria: 1) at least one blood, pleural fluid, or transthoracic needle aspiration culture positive for *S.pneumoniae*; 2) bacterial growth of ≥103 colony-forming units/milliliter (CFU/mL) of *S.pneumoniae* from a protected specimen brush, and/or ≥104 CFU/mL in bronchoalveolar lavage; 3) positive urinary antigen for *S.pneumoniae* with a diagnosis of probable pneumococcal pneumonia, using a commercially available immunochromatographic assay (Binax NOW).

**Statistical analysis**

The comparison of the distribution of genotypes based on the susceptibility, severity and outcome were performed with the χ^2^ test or Fisher exact test when needed, and odds ratios (OR) with 95% of confidence intervals (95% CI) were calculated. The relation between severity or outcome and genotypes was evaluated by binary logistic regression models, and age, gender, hospital of origin and co-morbidities, or PSI and pathogen were included as independent variables. Survival rates were estimated using the Kaplan-Meier method and their comparison related to genotypes was performed with log-rank test. Multivariate analysis adjusted for the independent variables was carried out with Cox proportional hazard model. Alleles and haplotypes were recoded in binary variables for dominant and recessive effects. Quantitative variables are presented using arithmetic mean ± SEM. The differences in serum levels with regard to the analyzed genotypes were compared with the U of Mann-Whitney, or with the H of Kruskal-Wallis when more than two genotypes were present. Statistical significance was taken as *P* value < 0.05. Bonferroni correction for multiple comparisons was applied when the frequencies of SNPs and haplotypes were compared between patients and controls.

**TABLES**

**Table E1. Resulting haplotypes from SNPs combination in *SFTPA1* and *SFTPA2* genes.**

| Haplotype | Nucleotide/amino acid | | | | |
| --- | --- | --- | --- | --- | --- |
| *SFTPA1* | aa19 | aa50 | aa62^*^ | aa133^*^ | aa219 |
| *6A* | C/Ala | C/Leu | G | G | C/Arg |
| *6A^2^* | T/Val | G/Val | A | A | C/Arg |
| *6A^3^* | T/Val | C/Leu | A | A | C/Arg |
| *6A^4^* | T/Val | C/Leu | G | A | T/Trp |
|  |  |  |  |  |  |
| *SFTPA2* | aa9 | aa91 | aa140^*^ | aa223 |  |
| *1A* | C/Thr | C/Pro | C | C/Gln |  |
| *1A^0^* | A/Asn | G/Ala | C | C/Gln |  |
| *1A^1^* | C/Thr | G/Ala | T | A/Lys |  |
| *1A^2^* | C/Thr | G/Ala | C | C/Gln |  |
| *1A^3^* | A/Asn | G/Ala | T | A/Lys |  |
| *1A^5^* | C/Thr | C/Pro | T | C/Gln |  |

SNPs: Single nucleotide polymorphisms.

Haplotypes are named as *6A^n^* for *SFTPA1* and *1A^n^* for *SFTPA2* based on previous nomenclature [15]. Only those haplotypes with a frequency higher than 1% are depicted.

^*^Nucleotide change that does not produce amino acid change.

**Table E2. D**emographic and clinical characteristics of CAP patients.

| Characteristics^*^ | N (%) |
| --- | --- |
| Age (682) | 62.95 ± 17.73^†^ |
| Sex (682) |  |
| Male | 435 (63.78) |
| Female | 247 (36.22) |
| ICU admission (682) |  |
| No (91.57±34.45 )^‡^ | 526 (77.13) |
| Yes (118.41±40.29 ) ^‡^ | 156 (22.87) |
| MODS (682) |  |
| No (92.59±34.33) ^‡^ | 593 (86.95) |
| Yes (133.68±39.86) ^‡^ | 89 (13.05) |
| ARDS (682) |  |
| No (96.67±37.23) ^‡^ | 656 (96.19) |
| Yes (122.48±37.67) ^‡^ | 26 (3.81) |
| 90-day exitus (682) |  |
| No (95.03±36.44) ^‡^ | 636 (93.26) |
| Yes (134.67±33.60) ^‡^ | 46 (6.74) |
| 28-day exitus (682) |  |
| No (95.70±36.66) ^‡^ | 648 (95.01) |
| Yes (136.71±34.16) ^‡^ | 34 (4.99) |
| Co-morbidity (670)^§^ |  |
| No | 226 (33.14) |
| COPD | 201 (30.00) |
| Asthma | 29 (4.33) |
| Neoplasy | 76 (11.34) |
| Ischemic cardiopathy | 87 (12.99) |
| Diabetes | 148 (22.09) |
| Renal insufficiency | 50 (7.46) |
| Hepatic insufficiency | 45 (6.72) |
| Neurological pathology | 90 (13.43) |
| Autoimmune pathology | 13 (1.94) |
| Psiquiatric pathology | 5 (0.75) |
| Pneumonia severity index (667) |  |
| I-III (low) | 290 (43.48) |
| IV-V (moderate-high) | 377 (56.52) |
| Etiological agent identified (682) |  |
| No | 408 (59.82) |
| Yes | 274 (40.18) |
| *Streptococcus pneumonia* | 163 (23.90) |
| *Pseudomonas aeruginosa* | 19 (2.79) |
| *Legionella ssp* | 13 (1.91) |
| *Haemophylus influenza* | 9 (1.32) |
| Virus | 11 (1.61) |
| Others | 59 (8.65) |

CAP: Community-acquired pneumonia; ICU: Intensive care unit; MODS: Multi-organ dysfunction syndrome; ARDS: Acute respiratory distress syndrome; COPD: Chronic obstructive pulmonary disease.

^*^In brackets the number of patients with available data.

^†^For age the value is mean ± standard deviation.

^‡^Pneumonia Severity Index (mean ± SD).

^§^Some patients had more than one co-morbidity.

**Table E3. Pairwise linkage disequilibrium measure (D’) for surfactant proteins A1, A2 and D alleles from 748 healthy controls.**

|  |  | ***SFTPA1^*^*** | | | | | | |  |
| --- | --- | --- | --- | --- | --- | --- | --- | --- | --- |
|  |  | ***6A*** | ***6A^2^*** | ***6A^3^*** | ***6A^4^*** | ***6A^5^*** | ***6A^12^*** | ***6A^15^*** | **SP-D** |
| ***SFTPA2^*^*** | ***1A*** | 0.34  (<0.0001) | 0.72  (<0.0001) | - | - | 1  (<0.0001) | - | 0.65  (<0.0001) | 0.31  (<0.0001) |
|  | ***1A^0^*** | 0.74  (<0.0001) | 0.40  (<0.0001) | 0.40  (<0.0001) | 0.61  (<0.0001) | - | - | - | - |
|  | ***1A^1^*** | - | 0.50  (<0.0001) | 0.51  (<0.0001) | - | - | - | - | - |
|  | ***1A^2^*** | - | 0.48  (<0.0001) | - | 0.37  (<0.0001) | - | - | - | - |
|  | ***1A^3^*** | - | - | - | - | - | - | - | - |
|  | ***1A^7^*** | - | - | - | - | - | - | - | - |
|  | ***1A^10^*** | - | - | - | - | - | - | - | - |
|  | ***1A^13^*** | - | - | - | - | - | - | - | - |
|  | **SP-D** | 0.93  (<0.0001) | - | - | - | - | 0.84  (0.006) | 0.71  (0.038) |  |

The numbers are D’ (*P* value). Those D’ values lower than 0.3, or with a corresponding *P* value higher than 0.05 have not been considered.

^*^Haplotypes for *SFTPA1* and *SFTPA2,* resulting from the different combinations of the three SNPs (Single nucleotide polymorphisms) studied at each gene, are denoted using the conventional nomenclature [15].

**Table E4. Comparison of haplotypes of *SFTPA1, SFTPA2* and *SFTPD* between patients with pneumococcal CAP and controls.**

| Haplotype^*^ | Controls  N=1538 | PCAP  N=326 | *P*^†^  OR (95%CI) |
| --- | --- | --- | --- |
| *SFTPA1* |  |  |  |
| *6A^2^* (*TGC)* | 934 (60.7) | 177 (54.3) | 0.032  0.77 (0.60-0.99) |
| *SFTPA2* |  |  |  |
| *1A^0^* (*AGC*) | 911 (59.2) | 169 (51.8) | 0.014  0.74 (0.58-0.95) |
| *1A^10^* (*CCA*) | 4 (0.3) | 5 (1.5) | 0.011^‡^  5.97 (1.28-30.23) |
| *SFTPA1-SFTP2* |  |  |  |
| *6A^2^-1A^0^* | 802 (52.1) | 147 (45.1) | 0.022  0.75 (0.59-0.96) |
| *SFTPC-SFTPA1-SFTP2* |  |  |  |
| *C-6A^3^-1A* | 3 (0.2) | 4 (1.2) | 0.021^‡^  6.36 (1.07-43.53) |

Frequency values are the number of chromosomes (%). PCAP: Pneumococcal community-acquired pneumonia. Only those haplotypes with significant differences between PCAP and healthy controls were included.

^*^Haplotypes for *SFTPA1* and *SFTPA2,* resulting from the different combinations of the three SNPs (Single nucleotide polymorphisms) studied at each gene, are denoted using the conventional nomenclature [15].

^†^*P* value for the bivariate comparison.

^‡^*P* value by Fischer exact test.
